# Supplementary material for: Spearfishing and public health promotion: A cross-sectional analysis of the Hawaiʻi Behavioral Risk Factor Surveillance System Survey
Source: PLoS One. 2025 Mar 21;20(3):e0319169. doi: 10.1371/journal.pone.0319169 (PMC11927901; doi:10.1371/journal.pone.0319169)
Supplement: S1 File — (DOCX) [file pone.0319169.s001.docx]

**Supporting Information: Variable Categorization**

This supplemental document provides a detailed description and rationale for how variables in this spearfishing engagement study were categorized based on the variables in the BRFSS dataset. It also provides the variable names that correspond with the limited dataset that accompanies this manuscript.

**Demographic Variables**

**Sex**

Left as a binary variable according to the BRFSS survey, removing any missing values: Male and Female.

**Age**

Binned into categories (18–24, 25–34, 35–44, 45–54, 55–64, ≥65 years) from a continuous age variable, based on the categorization in the Sentell et al. hula and paddling study (1).

Age was grouped to allow for comparisons across life stages, consistent with the Sentell et al. paper, which highlighted differences in engagement in culturally relevant physical activities across age groups (1). Grouping helps identify key trends in participation and health outcomes associated with spearfishing among younger and older adults.

**Race/Ethnicity**

Used Hawai‘i State Department of Health standards for race/ethnicity categories, which offer regionally relevant classifications, including Native Hawaiians, other Pacific Islanders, and subcategories of Asian ethnicities to allow for more nuanced comparisons (2)

**Education**

The BRFSS survey categorizes education levels as follows: Never attended school or only kindergarten; Grades 1–8 (Elementary); Grades 9–11 (Some High School); Grade 12 or GED (High School Graduate); College 1–3 years (Some College or Technical School); College 4 years or more (College Graduate); and Refused or Missing (3). For simplicity, and due to low response counts in the lower education categories, these were grouped into broader categories: Less than High School or High School Diploma; High School or Some College; and College Graduate.

**Income**

Income levels were categorized into ranges consistent with Sentell et al. to facilitate comparability with prior research on culturally relevant physical activities in Hawai‘i. These categories were as follows: <$15,000; $15,000–$24,999; $25,000–$49,999; $50,000–$74,999; $75,000–$124,999; ≥$125,000 (1).

This approach ensures alignment with previous studies, as well as allowing for meaningful analysis of socioeconomic disparities in health outcomes and engagement in spearfishing.

**Marital Status**

Binned as a binary outcome: Married/Partnered or Single. For the sake of our study, we assume that all divorced and widow/er respondents are not currently partnered.

**Health Status Variables**

**General Health Status**

From the general health status data, responses were categorized into excellent, very good, good, fair, and poor (3). To account for above-average and below-average general health outcomes, very good and excellent, and poor and fair were each binned into individual categories.

**BMI**

Kept in the original BRFSS categories, calculated from BMI: Underweight, Normal weight, Overweight, Obese.

**Health-Related Variables**

The BRFSS survey treats many health status variables as Yes or No outcomes, with a Missing/Don’t Know/Refused category. For our analysis, these were all binned into binary variables, with any missing responses removed from the analysis.

- Smoking Status
- Difficulty dressing or bathing
- Difficulty walking or climbing up stairs
- Arthritis
- Asthma
- Visited a doctor in the last 12 months
- Diabetes
- Hypertension
- Physical activity
- Depression
- Heart disease
- Health insurance

In the case of hypertension and visiting a doctor in the last 12 months, additional response categories were consolidated. For hypertension, those who had been told they were hypertensive but only while pregnant and those who were told they were borderline hypertensive were categorized as not having hypertension.

The correlate of *visiting a doctor in the last 12 months* was adapted from the BRFSS survey question: *How long has it been since you last visited a doctor for a routine checkup?* With the respective responses including: being within the past year, within the past 2 years, within the past 5 years, 5 or more years ago, Don’t Know/Not Sure, Never, Refused (3). This was consolidated into a binary outcome, as 80% of respondents reported visiting a doctor in the last year.

**Household Characteristics**

**Number of Children**

Family size was binned from a continuous variable into categories: 0, 1, 2, 3, and ≥ 4 children.

**Spearfishing Engagement**

Spearfishing engagement was originally reported in the BRFSS survey using five response categories: Never, Almost Never, Sometimes, Often, and Very Often. Due to the subjective nature of these categories and to ensure meaningful analysis, the variable was dichotomized to: sometimes/often/very often" and almost never/never. This recoding allows for direct comparison with the Sentell et al. paper that examined hula and paddling prevalence (1). S2 provides study results using another coding of this variable, dichotomizing to often/very often and sometimes/almost never/never.

**Additional Information**

Any additional inquiries on the variable descriptions not detailed here can be accessed in the BRFSS codebooks for 2019 and 2020, respectively. Please note that these are the codebooks for national surveys and do not include spearfishing engagement or the Hawaiʻi State Department of Health race categories mentioned in our study.

<https://www.cdc.gov/brfss/annual_data/2019/pdf/codebook19_llcp-v2-508.HTML>

<https://www.cdc.gov/brfss/annual_data/2020/pdf/codebook20_llcp-v2-508.pdf>

**Variable Names**

**Literature Cited**

1. Sentell T, Wu YY, Look M, Gellert K, Lowery St John T, Ching L, et al. Culturally Relevant Physical Activity in the Behavioral Risk Factor Surveillance System in Hawai’i. Prev Chronic Dis. 2023 May 25;20:E43.

2. HHDW. 2022 Race-Ethnicity Documentation [Internet]. Hawaiʻi Health Data Warehouse; 2022 [cited 2024 Jan 12]. Available from: https://hhdw.org/wp-content/uploads/2022/04/Race-Ethnicity_4.2.22.pdf

3. Centers for Disease Control and Prevention (CDC). Behavioral Risk Factor Surveillance System Survey Questionnaire. U.S. Department of Health and Human Services, Centers for Disease Control and Prevention; 2021.
