# Supplementary material for: Spearfishing and public health promotion: A cross-sectional analysis of the Hawaiʻi Behavioral Risk Factor Surveillance System Survey
Source: PLoS One. 2025 Mar 21;20(3):e0319169. doi: 10.1371/journal.pone.0319169 (PMC11927901; doi:10.1371/journal.pone.0319169)
Supplement: S1 Table — (DOCX) [file pone.0319169.s003.docx]

Table S1: Prevalence of spearfishing (Often/Very Often), by demographic and health characteristics, among participants in the 2019 and 2020 H-BRFSS

|  | |  |  | **Model 1** |  | **Model 2** |  | **Model 3** |  |
| --- | --- | --- | --- | --- | --- | --- | --- | --- | --- |
|  | **Prevalence (%) and 95% CI** | **cPR (95%)** | **p-value** | **aPR (95% CI)** | **p-value** | **aPR (95% CI)** | **p-value** | **aPR (95% CI)** | **p-value** |
| **Total Sample** | 10.1 (9.4,10.8) |  |  |  |  |  |  |  |  |
| **Sex** |  |  |  |  |  |  |  |  |  |
| Female | 2.5 ( 2.0, 3.1) | Ref |  |  |  |  |  |  |  |
| Male | 17.5 (16.3,18.9) | 7.02 (5.62, 8.77) | <0.0001 | 7.07 (5.67, 8.81) | <0.0001 | 6.89 (5.52, 8.59) | <0.0001 |  |  |
| **Age Group (years)** |  |  |  |  |  |  |  |  |  |
| 18-24 | 12.6 (9.8,16.2) | Ref |  |  |  |  |  |  |  |
| 25-34 | 11.1 (9.3,13.2) | 0.88 (0.64, 1.19) | 0.3993 | 0.89 (0.67, 1.18) | 0.4134 | 0.85 (0.64, 1.13) | 0.2574 | 0.81 (0.61, 1.08) | 0.1523 |
| 35-44 | 10.7 (8.9,12.8) | 0.85 (0.62, 1.15) | 0.2922 | 0.95 (0.72, 1.26) | 0.7239 | 0.88 (0.66, 1.17) | 0.3712 | 0.82 (0.62, 1.09) | 0.1704 |
| 45-54 | 9.3 (7.8,11.1) | 0.74 (0.55, 1.00) | 0.0533 | 0.81 (0.61, 1.08) | 0.1575 | 0.76 (0.57, 1.01) | 0.0594 | 0.70 (0.52, 0.93) | 0.0156 |
| 55-64 | 8.9 (7.6,10.5) | 0.71 (0.52, 0.95) | 0.0229 | 0.85 (0.65, 1.12) | 0.2539 | 0.78 (0.59, 1.03) | 0.0841 | 0.71 (0.53, 0.96) | 0.0236 |
| 65+ | 9.2 (8.0,10.5) | 0.73 (0.55, 0.97) | 0.0302 | 1.02 (0.78, 1.32) | 0.9094 | 0.87 (0.67, 1.14) | 0.3245 | 0.79 (0.58, 1.05) | 0.1075 |
| **Race/Ethnicity** |  |  |  |  |  |  |  |  |  |
| White | 6.0 ( 5.1, 7.0) | Ref |  |  |  |  |  |  |  |
| Native Hawaiian | 21.5 (19.3,23.9) | 3.61 (2.98, 4.38) | <0.0001 | 3.89 (3.21, 4.72) | <0.0001 | 3.95 (3.26, 4.80) | <0.0001 | 3.82 (3.13, 4.66) | <0.0001 |
| Other Pacific  Islander | 16.4 (12.5,21.2) | 2.75 (2.02, 3.75) | <0.0001 | 3.10 (2.29, 4.18) | <0.0001 | 3.13 (2.32, 4.22) | <0.0001 | 2.95 (2.18, 3.99) | <0.0001 |
| Japanese | 10.5 ( 8.9,12.5) | 1.77 (1.40, 2.24) | <0.0001 | 2.15 (1.71, 2.70) | <0.0001 | 2.23 (1.77, 2.80) | <0.0001 | 2.21 (1.76, 2.78) | <0.0001 |
| Chinese | 4.8 ( 3.1, 7.5) | 0.81 (0.51, 1.29) | 0.3840 | 1.03 (0.65, 1.64) | 0.8919 | 1.11 (0.70, 1.75) | 0.6566 | 1.13 (0.72, 1.77) | 0.6056 |
| Filipino | 6.1 ( 4.8, 7.8) | 1.03 (0.77, 1.38) | 0.8422 | 1.30 (0.97, 1.73) | 0.0762 | 1.39 (1.05, 1.86) | 0.0230 | 1.38 (1.03, 1.84) | 0.0283 |
| Other Asian | 5.6 ( 3.7, 8.3) | 0.93 (0.60, 1.45) | 0.7597 | 1.20 (0.78, 1.85) | 0.4033 | 1.23 (0.80, 1.91) | 0.3470 | 1.24 (0.80, 1.92) | 0.3312 |
| American Indian  or Alaskan Native | 12.5 ( 7.4,20.4) | 2.10 (1.23, 3.58) | 0.0065 | 1.93 (1.18, 3.14) | 0.0086 | 1.93 (1.18, 3.15) | 0.0086 | 1.87 (1.15, 3.05) | 0.0123 |
| Black | 2.7 ( 0.9, 7.5) | 0.45 (0.16, 1.30) | 0.1408 | 0.49 (0.17, 1.40) | 0.1851 | 0.51 (0.18, 1.44) | 0.2004 | 0.49 (0.17, 1.38) | 0.1769 |
| Other | 6.9 ( 3.7,12.6) | 1.16 (0.62, 2.18) | 0.6384 | 1.08 (0.59, 1.98) | 0.7963 | 1.11 (0.61, 1.99) | 0.7371 | 1.10 (0.62, 1.97) | 0.7379 |
| **Education Level** |  |  |  |  |  |  |  |  |  |
| College  Graduate | 6.7 ( 5.9, 7.6) | Ref |  |  |  |  |  |  |  |
| Some College | 9.7 ( 8.6,11.0) | 1.45 (1.21, 1.73) | <0.0001 | 1.23 (1.03, 1.47) | 0.0219 | 1.19 (1.00, 1.42) | 0.0500 | 1.19 (1.00, 1.41) | 0.0534 |
| High School  Diploma or Less | 13.3 (11.9,14.9) | 1.99 (1.68, 2.36) | <0.0001 | 1.42 (1.19, 1.70) | <0.0001 | 1.36 (1.14, 1.63) | 0.0006 | 1.34 (1.12, 1.60) | 0.0012 |
| **Federal Poverty Level** |  |  |  |  |  |  |  |  |  |
| 0-130% | 10.9 (9.4,12.6) | Ref |  |  |  |  |  |  |  |
| 131-185% | 10.6 (8.7,13.0) | 0.98 (0.76, 1.25) | 0.8484 | 1.10 (0.87, 1.38) | 0.4297 | 1.13 (0.90, 1.42) | 0.2932 | 1.12 (0.89, 1.41) | 0.3309 |
| 186+% | 9.8 (8.9,10.8) | 0.90 (0.76, 1.07) | 0.2513 | 1.14 (0.95, 1.36) | 0.1525 | 1.13 (0.95, 1.35) | 0.1614 | 1.14 (0.95, 1.36) | 0.1572 |
| Don’t know/refused | 8.9 (6.7,11.7) | 0.82 (0.60, 1.12) | 0.2120 | 0.92 (0.70, 1.22) | 0.5855 | 0.94 (0.71, 1.24) | 0.6729 | 0.94 (0.72, 1.24) | 0.6614 |
| **Island of Residence** |  |  |  |  |  |  |  |  |  |
| Oʻahu | 8.5 ( 7.7, 9.5) | Ref |  |  |  |  |  |  |  |
| Hawaiʻi Island | 13.6 (11.8,15.5) | 1.59 (1.34, 1.89) | <0.0001 | 1.51 (1.28, 1.79) | <0.0001 | 1.50 (1.27, 1.78) | <0.0001 | 1.51 (1.28, 1.79) | <0.0001 |
| Kauai | 13.0 (10.5,15.9) | 1.52 (1.21, 1.92) | 0.0004 | 1.64 (1.35, 2.00) | <0.0001 | 1.63 (1.34, 1.98) | <0.0001 | 1.66 (1.36, 2.02) | <0.0001 |
| Maui | 12.1 (10.2,14.2) | 1.42 (1.17, 1.72) | 0.0004 | 1.47 (1.22, 1.76) | <0.0001 | 1.45 (1.21, 1.74) | <0.0001 | 1.46 (1.22, 1.75) | <0.0001 |
| Molokai | 28.7 (19.3,40.3) | 3.37 (2.30, 4.93) | <0.0001 | 2.70 (2.05, 3.57) | <0.0001 | 2.51 (1.90, 3.32) | <0.0001 | 2.51 (1.89, 3.32) | <0.0001 |
| Lanai | 32.7 (19.0,50.0) | 3.83 (2.34, 6.27) | <0.0001 | 3.42 (2.13, 5.48) | <0.0001 | 3.16 (1.95, 5.14) | <0.0001 | 3.10 (1.92, 5.00) | <0.0001 |
| **Marital Status** |  |  |  |  |  |  |  |  |  |
| Married or  Partnered | 10.0 (9.1,11.0) | Ref |  |  |  |  |  |  |  |
| Single | 10.1 (9.1,11.3) | 1.01 (0.87, 1.17) | 0.9034 | 0.93 (0.80, 1.07) | 0.2923 | 0.92 (0.80, 1.06) | 0.2554 | 0.93 (0.81, 1.06) | 0.2755 |
| **Ever smoked 100 cigarettes** |  |  |  |  |  |  |  |  |  |
| No | 7.9 ( 7.1, 8.8) | Ref |  |  |  |  |  |  |  |
| Yes | 13.7 (12.4,15.0) | 1.73 (1.50, 1.99) | <0.0001 |  |  | 1.36 (1.19, 1.57) | <0.0001 | 1.36 (1.18, 1.56) | <0.0001 |
| **Met Physical Activity Guidelines** |  |  |  |  |  |  |  |  |  |
| Did not meet | 7.0 ( 5.7, 8.5) | Ref |  |  |  |  |  |  |  |
| Met | 11.9 (10.5,13.4) | 1.70 (1.35, 2.14) | <0.0001 |  |  | 1.65 (1.33, 2.04) | <0.0001 | 1.69 (1.36, 2.09) | <0.0001 |
| 2020/Don’t  know/refused | 10.4 ( 9.4,11.4) | 1.48 (1.19, 1.84) | 0.0004 |  |  | 1.42 (1.16, 1.75) | 0.0007 | 1.07 (0.65, 1.75) | 0.7894 |
| **Visited a doctor in the last 12 months** | |  |  |  |  |  |  |  |  |
| No | 11.1 (9.5,12.8) | Ref |  |  |  |  |  |  |  |
| Yes | 9.8 (9.1,10.7) | 0.89 (0.75, 1.06) | 0.1805 |  |  | 1.12 (0.95, 1.33) | 0.1874 | 1.09 (0.92, 1.30) | 0.3073 |
| **BMI Categories** |  |  |  |  |  |  |  |  |  |
| Normal | 6.9 ( 6.0, 8.0) | Ref |  |  |  |  |  |  |  |
| Overweight | 11.2 (10.0,12.4) | 1.61 (1.34, 1.93) | <0.0001 |  |  |  |  | 1.12 (0.95, 1.33) | 0.1831 |
| Obese | 14.2 (12.5,16.0) | 2.05 (1.69, 2.47) | <0.0001 |  |  |  |  | 1.23 (1.02, 1.49) | 0.0298 |
| Underweight | 4.4 ( 2.3, 8.2) | 0.63 (0.33, 1.21) | 0.1695 |  |  |  |  | 0.80 (0.44, 1.46) | 0.4731 |
| **Self Rated Health** |  |  |  |  |  |  |  |  |  |
| Excellent/Very  Good | 9.1 ( 8.2,10.1) | Ref |  |  |  |  |  |  |  |
| Good | 10.8 ( 9.6,12.1) | 1.18 (1.01, 1.39) | 0.0424 |  |  |  |  | 0.94 (0.81, 1.10) | 0.4575 |
| Fair/poor | 12.1 (10.3,14.2) | 1.33 (1.09, 1.61) | 0.0042 |  |  |  |  | 0.96 (0.79, 1.16) | 0.6425 |
| **Depressive Disorder** |  |  |  |  |  |  |  |  |  |
| No | 10.3 (9.5,11.1) | Ref |  |  |  |  |  |  |  |
| Yes | 8.9 (7.3,10.7) | 0.86 (0.70, 1.06) | 0.1580 |  |  |  |  | 0.94 (0.77, 1.15) | 0.5495 |
| **Diabetes** |  |  |  |  |  |  |  |  |  |
| No | 9.7 ( 9.0,10.5) | Ref |  |  |  |  |  |  |  |
| Yes | 13.1 (10.9,15.7) | 1.36 (1.11, 1.66) | 0.0027 |  |  |  |  | 1.07 (0.87, 1.30) | 0.5328 |
| **High Blood Pressure** |  |  |  |  |  |  |  |  |  |
| No | 9.3 (8.2,10.6) | Ref |  |  |  |  |  |  |  |
| Yes | 10.7 (9.0,12.5) | 1.15 (0.93, 1.41) | 0.1963 |  |  |  |  | 0.87 (0.72, 1.07) | 0.1823 |
| 2020/Don’t  Know/Refused | 10.4 (9.4,11.5) | 1.12 (0.95, 1.32) | 0.1713 |  |  |  |  | 1.29 (0.81, 2.07) | 0.2866 |
| **Heart Disease** |  |  |  |  |  |  |  |  |  |
| No | 9.9 ( 9.2,10.7) | Ref |  |  |  |  |  |  |  |
| Yes | 15.4 (10.7,21.7) | 1.56 (1.09, 2.24) | 0.0160 |  |  |  |  | 1.14 (0.84, 1.55) | 0.3860 |
| **Arthritis** |  |  |  |  |  |  |  |  |  |
| No | 9.9 (9.1,10.7) | Ref |  |  |  |  |  |  |  |
| Yes | 10.7 (9.2,12.4) | 1.08 (0.91, 1.28) | 0.3653 |  |  |  |  | 1.11 (0.94, 1.30) | 0.2246 |
| **Asthma** |  |  |  |  |  |  |  |  |  |
| No | 9.9 (9.1,10.7) | Ref |  |  |  |  |  |  |  |
| Yes | 11.1 (9.3,13.2) | 1.12 (0.92, 1.36) | 0.2478 |  |  |  |  | 1.05 (0.88, 1.25) | 0.5882 |
| **Difficulty Walking or Climbing up stairs** | |  |  |  |  |  |  |  |  |
| No | 9.9 (9.2,10.7) | Ref |  |  |  |  |  |  |  |
| Yes | 11.5 (9.4,14.1) | 1.16 (0.94, 1.44) | 0.1662 |  |  |  |  | 1.25 (1.01, 1.56) | 0.0438 |
| **Difficulty Dressing or bathing** |  |  |  |  |  |  |  |  |  |
| No | 10.0 (9.3,10.8) | Ref |  |  |  |  |  |  |  |
| Yes | 11.6 (8.1,16.3) | 1.16 (0.81, 1.65) | 0.4249 |  |  |  |  | 0.76 (0.55, 1.07) | 0.1131 |
